# Exploiting Identifiability and Intergene Correlation for Improved Detection of Differential Expression

**DOI:** 10.1155/2013/404717

**Published:** 2013-06-03

**Authors:** J. R. Deller, Hayder Radha, J. Justin McCormick

**Affiliations:** ^1^Department of Electrical and Computer Engineering, Michigan State University, 2120 EB, East Lansing, MI 48824, USA; ^2^Department of Molecular Biology & Biochemistry, Carcinogenesis Laboratory, Michigan State University, 341 FST, East Lansing, MI 48824, USA

## Abstract

Accurate differential analysis of microarray data strongly depends on effective treatment of intergene correlation. Such dependence is ordinarily accounted for in terms of its effect on significance cutoffs. In this paper, it is shown that correlation can, in fact, be exploited to share information across tests and reorder expression differentials for increased statistical power, regardless of the threshold. Significantly improved differential analysis is the result of two simple measures: (i) adjusting test statistics to exploit information from identifiable genes (the large subset of genes represented on a microarray that can be classified *a priori* as nondifferential with very high confidence], but (ii) doing so in a way that accounts for linear dependencies among identifiable and nonidentifiable genes. A method is developed that builds upon the widely used two-sample *t*-statistic approach and uses analysis in Hilbert space to decompose the nonidentified gene vector into two components that are correlated and uncorrelated with the identified set. In the application to data derived from a widely studied prostate cancer database, the proposed method outperforms some of the most highly regarded approaches published to date. Algorithms in MATLAB and in R are available for public download.

## 1. Preamble

In certain ways, this paper represents a departure from current trends in scientific publishing. The Worldwide Web has made available extraordinary resources in the form of databases for comparative analysis of methods in bioinformatics and numerous other disciplines. The benefits of using *common* sets of *real* data to compare and contrast new algorithms are obvious. In some fields of investigation, especially, perhaps, research in early states of knowledge (e.g., genomics), there is an equally obvious drawback in using real data—that the “correct answers are not known,” making it difficult to ultimately interpret differences in performance as anything but differences.

Lest the reader be preparing for an argument promoting classic simulation studies, we hasten to state at the outset that this argument is not forthcoming. Before the age of the internet, simulation studies using reasonably justified data models (Gaussian errors, etc.) were a time-honored standard in all areas of math, science, and engineering. The ready availability of rich data resources makes it irrational to advocate to a return to “pure simulation” using models that are untested against these existing data sets. The authors of this paper in no way promote a return to such methods and appeal to the reader to recognize that “models detached from reality” are not used in any way in this paper.

This research centers on some rather straightforward adjustments to classic hypothesis-testing procedures for use in differential analysis of microarray expression data. The salient result is a “reranking” of the order in which gene expression data are considered for “truly differential” status. In an effort to objectively compare these modified methods with the performance of established algorithms, it was decided to create a database of microarray expression simulations in which the data retained as much second-order statistical character as possible relative to a widely used prostate cancer database. The up- and downregulations of gene expressions were indeed synthetically modulated onto a carefully constructed baseline. The consequence is a set of comparative performance results that are objectively based on data that were “guided by nature” but which were, quite openly stated, synthesized with this natural guidance. Rather than dismissing these results as “simulations,” the reader is urged to consider whether there is merit in moving the uncertainty to the data generation side (if that uncertainty can be intelligently controlled), if it permits objective results on the data analysis side. The current *modus operandi* is to accept uncertainty in the performance results with the benefit of authenticity in the data generation. The authors hope that this question might engender some debate and research.

Using the testing approach employing “nature-guided simulation,” the results for the reranking method presented here are remarkably good relative to two established methods developed by respected authorities. Many people have reviewed these results, including some very eminent statisticians and bioinformaticians. Reactions have ranged from encouragement and amazement to deep skepticism. The comment “too good to be true” has been used at least twice, including, once, in a constructive criticism, by a distinguished editor of this journal. We understand this response: unfortunately (or fortunately, depending on one's perspective), no one has been able to find a flaw in the methods. We suggest two possibilities. (1) Some aspect of the simulation procedure is creating a bias toward the developed detection method. (2) Authors with a somewhat different perspective (two signal processing engineers and a cancer researcher, with over a century of combined research experience, but without extensive work in bioinformatics) were able to see some relatively simple algorithmic adjustments that eluded researchers focusing on deeper issues.

Rather than viewing the results of this paper as a claim of superiority of the new method over respected algorithms, the authors appeal to the reader to accept the report in the spirit it is offered: interesting, potentially useful, results that raise many questions, and possibilities for further research. The “intelligent stimulation” approach in itself may offer some grounds for innovation in the field. Attempts to verify that the present results are, indeed, “too good to be true” may reveal technical information benefiting differential expression detection methods. This is the classical way in which research moves forward. We are grateful to the *ISRN Journal of Bioinformatics* for the opportunity to bring these ideas to the attention of the research community.

## 2. Introduction

 The DNA microarray was initially touted as a tool that would revolutionize the understanding of complex diseases and usher in an era of personalized medicine. This optimism is on display in Lander's 1999 *Nature Genetics* article entitled “Array of hope” [1]. It is not unusual, however, for near-term impacts of emerging technologies to be overestimated when first deployed, then to have the expectations moderated as the technologies reveal new complexities in the problems they are designed to solve. Over the past decade, early optimism about the microarray has given way to a pragmatic understanding of challenges and the need for further research and development. This normal course of events led to Frantz's 2005 article in *National Review of Drug Discovery* entitled “An array of problems” [2]. The study of microarray data has shown the need for exceeding care in the design and regularization of experiments and in the data collection and preprocessing, but the biggest hindrance to progress has been the lack of definitive methods for *interpretation* of microarray results.

One of the main challenges to proper analysis is the presence of significant correlation among gene expressions manifest in the microarray results [3, 4]. One measurable indication of the uncertainty caused by correlated differential-expression tests is the resultant increase in the variance of the *false discovery rate* (FDR) [3]. Linear statistical dependence among gene-expression correlations, therefore, can be quantifiably linked to higher-risk detection algorithms for the discovery of active genes. Among many causes, intergene correlation is attributable to coexpression of genes [5] and to unmodeled factors that introduce systematic effects across genes [6, 7]. As a result, for most real data, the assumption of independence or weak dependence among gene expressions is unfounded, and methods treating correlation are necessary [8, 9]. In fact, a few investigators have even questioned the adequacy of accounting for correlation alone and have examined the implications of nonlinear dependence on the discovery of genes [10–12].

Correctly detecting differentially expressed genes—or the related task of estimating the FDR—in the presence of substantial intergene correlation is a challenging problem that has received much recent attention since reported in papers by Owen, Efron, and others (e.g., [3, 4, 9, 13]). For example, Storey et al. [14] present an approach to the notion of sharing information across *t* scores, which they describe as “borrowing strength across the tests” for a potential increase in statistical power. Tibshirani and Wasserman [15] discuss a quantity called the “correlation-shared” *t*-statistic and derive theoretical bounds on its performance. Hu et al. [16] examine the covariance structure of the expression data and discover benefits of linking coexpression and differential expression in a distance measure—reflecting the more recent interest in characterizing broader statistical patterns in microarray data.

Recent research that is jointly concerned with differential expression and coexpression has also yielded results and methods that could ultimately benefit the gene discovery problem. Because the differential coexpression research is often concerned with differing phenotypes, rather than with different treatment conditions, two given research efforts involving differential coexpression might seek answers to different sets of genetic questions through expression data. Like the “treatment conditions researchers,” however, the “phenotype” researchers have encountered their own forms of confounding dependencies, notably the relative gene locations, the expression time sequencing, and phase information (e.g., [17–19]). Papers have been published addressing these issues, including the exposition of new statistical approaches—for example, “CorScor” [20], the “ECF-statistic” [21], gene-set coexpression analysis [22], fuzzy expression level assignments [23], expression-profile mining with decorrelation [24], and detection of microarray outliers [25]—as well as new clustering methods—for example, a web-based expression analyzer [26], high-order preclustering methods [27], and the “BioSym” distance measure [28]. A recent review of clustering methods in genomics appears in the paper by Dalton et al. [29]. A more general examination of the performance of classifiers of microarray expressions appears in the paper by Ancona et al. [30].

This paper is focused exclusively on the differential expression problem. Research in this area has largely focused on understanding harmful correlation effects on the choice of the threshold demarcating the statistical boundary between differential and nondifferential expression. In fact, however, the nominally confounding correlation can be used to advantage in increasing statistical power of microarray studies. This paper presents a differential analysis method that exploits identifiability and uses a gene expression reranking criterion that accounts for intergene correlation. The framework is readily generalizable for use in studies with multiple or continuous covariates, as well as to other multiple comparison applications. An example method presented here builds upon the widely used two-sample *t*-statistic approach with a decomposition of the expression vectors into subspaces of correlated and uncorrelated components.

## 3. Problem Formulation

Suppose that expression data for *G* genes are measured on *M* microarrays, resulting in a gene expression matrix, say **X** ∈ ℝ^*G*×*M*^, with (*g*, *m*) element *x*_*gm*_. Each of the *M* microarray experiments takes place under one of two conditions (indexed by *k* = 1   or   2) such as control and treatment. These two subsets of the data are called *treatment groups* in the paper.

Based on the gene expression matrix, **X**, we seek to identify a “small” number, *G*_∗_ ≪ *G*, of genes that are significantly differentially expressed between the two groups. One widely used strategy (e.g., [31, 32]) is to hypothesize that each gene is *not* differentially expressed. We refer to this as the *null hypothesis*, denoted *ℍ*_0_. For convenience, we use the shorthand notation *g* ∈ *ℍ*_0_ to indicate that the null hypothesis is known to be true for gene *g*, and, conversely, *g* ∉ *ℍ*_0_ indicates that gene *g* does not satisfy *ℍ*_0_. Gene *g* is tested against *ℍ*_0_ using a two-sample *t*-statistic, say, *t*_*g*_. The magnitudes of the statistics *t*_1_, *t*_2_,…, *t*_*G*_, establish a gene ranking and the *G*_∗_ genes with the largest *t*-scores are reported as statistically significant discoveries. The investigator can either supply a value for *G*_∗_ or rely on an estimation of the number of false discoveries (type I errors, false positives), say *𝔉*, or, equivalently, the FDR, defined as $\bar{𝔉}\overset{\mathrm{def}}{=}𝔉/G_{*}$, to find a maximal *G*_∗_ with the allowable *𝔉* or $\bar{𝔉}$ (e.g., [3, 5, 9, 13, 33–36]).

As discussed in [4, 11], for an “overpowered” **X** matrix, there may be significantly fewer tail-area null counts than expected, whereas for an “underpowered” **X**, the situation can worsen with an excessive number of tail-area null counts. It is important to note that techniques for estimating the FDR change the numbers of genes in reported lists, not the gene *rankings*. The present research was motivated by the hypothesis that, for an “underpowered” **X**, it would be possible to exploit correlation across *t* scores to establish a gene ranking with more statistical power than the raw *t*-based ranking. The method that resulted from an exploration of this question indeed seems to improve the statistical power of *all* **X** matrices.

The new method uses a vector of *t*-statistics,

(1) $$\begin{array}{c} t={\left[\begin{array}{cccc} t_{1} & t_{2} & \cdots & t_{G} \end{array}\right]}^{T}, \end{array}$$

and an estimate of the covariance matrix of the vector **t**, to output a substantially revised version of **t**, denoted **τ**, the entries of which provide an improved gene ranking. For expediency, we will refer to the procedure that produces **τ** from **t** as *correlation adjusted reranking*, or simply *reranking*. The essence of reranking is embodied in some fundamental data conditioning procedures to effect the two outcomes mentioned in the introduction—exploiting identifiability, and nullifying the effects of intergene correlation between identified and non-identified genes.

In the following sections, we develop the theoretical basis for the reranking process. The performance of reranking is then compared with that of state-of-the-art methods on data derived from real expression experiments. Results of some judiciously developed simulation studies are also reported for their value in understanding certain aspects of the performance.

## 4. Methods

### 4.1. Per Gene Summary Statistic 

Let us first supply the details surrounding the vector of *t* statistics introduced above. **t** will be viewed as a random vector with mean vector ***μ*** ∈ ℝ^*G*^ and covariance matrix Σ ∈ ℝ^*G*×*G*^. Henceforth, we write **t** ~ (***μ***, Σ). This is a theoretical model only, as ***μ*** and Σ are generally unknown. No further distribution information is required.

Let ${\bar{x}}_{g}$ denote the average differential expression level for gene *g*, ${\bar{x}}_{g}=M^{-1}\sum_{m=1}^{M}x_{gm}$. Further, let ${\bar{x}}_{g\;|\;k}$ be the average expression level for gene *g* in treatment condition *k*. Then, the (unpaired) *t*-statistic for gene *g* is computed as

(2) $$\begin{array}{c} t_{g}=\frac{{\bar{x}}_{g\;\;|\;\;2}-{\bar{x}}_{g\;\;|\;\;1}}{s_{g}}, \end{array}$$

where *s*_*g*_ is the pooled within-group sample standard deviation of gene *g*. If *g* ∈ *ℍ*_0_, then we expect *t*_*g*_ ~ (0, *ν*[*ν*−2]^−1^), where *ν* is the number of degrees of freedom, obtained from either the unpaired *t*-test theory or the permutation null calculations [4]. Otherwise, we expect *t*_*g*_ ~ (*μ*_*g*_, *σ*_*g*_^2^) with *μ*_*g*_ and *σ*_*g*_^2^ denoting the *g*th element of ***μ*** and the *g*th diagonal element of Σ, respectively. For *g* ∉ *ℍ*_0_, *μ*_*g*_ and *σ*_*g*_^2^ depend on the amount of up- or downregulation of the gene expression, the number of samples in each treatment group, and the number of degrees of freedom, *ν*.

### 4.2. Invoking Identifiability: The Zero Assumption

The direct use of *t*-scores for ranking neglects some important information that is inherent in the microarray matrix **X**. Fundamentally, the use of raw *t*-scores does not exploit *identifiability*, the strongly justified assumption that certain genes almost surely satisfy *ℍ*_0_. Formal backing for the creation of an identifiable set is found in Efron's *zero assumption* (ZA) [5], which states that a fraction, say *p*_0_, of the genes—those with the smallest *t*_*g*_ statistics—satisfies *ℍ*_0_. The ZA plays a central role in the literature on estimating the proportion of null genes, as in [13, 37]. The ZA is equally crucial for the two-group model approach developed in the Bayesian microarray literature, as in [38–40]. Furthermore, the assertion that the method developed by Storey [35] improves upon the well-known Benjamini-Hochberg FDR procedure [33] (in terms of statistical power) crucially relies on an adaptive version of the ZA.

The use of the ZA is justified in the reranking procedure as long as the parameter *p*_0_ is sufficiently small. For example, we set *p*_0_ ≈ 0.5 in experiments below based on the almost certain knowledge that ~50% of the genes in a cell would not be differentially expressed in the formation of prostate cancer [5]. Accordingly, in the initial step of the reranking process, we invoke the ZA to partition the *G* genes into an *identified set* of *G*_0_ elements assumed to satisfy *ℍ*_0_ and a *candidate set* of $G_{1}\overset{\mathrm{def}}{=}(G-G_{0})$ genes, so-named because they remain “candidates” to become “discovered” genes (i.e., to be among the *G*_∗_ ≤ *G*_1_ < *G* genes declared to be differentially expressed). For convenience and without any significant loss of generality (especially for large *G*), we will assume that if the fraction, *p*_0_, rather than the cardinality, *G*_0_, is used to specify the size of the identified set, then *p*_0_ is selected so that *p*_0_ *G* is an integer. That integer is therefore *G*_0_, and *p*_0_ = *G*_0_/*G*.

A simple corollary to the ZA is that the *t*_*g*_ value for each *g* ∈ *ℍ*_0_ represents a “noise” value in the *t*-score for that gene. This is because the expected value of this statistic is *ℰ*{*t*_*g*_} = 0 whenever *g* ∈ *ℍ*_0_. Accordingly, we can view the *t*_*g*_ value for *g* ∈ *ℍ*_0_ as a random variation around the nominal value of zero differential expression. The reranking procedure exploits this information to adjust the values of the candidate gene statistics. This “adjustment” is a consequence of decorrelating candidate expression values from those in the identified set. That these two subsets would be correlated may, at first, seem counterintuitive because the “interesting” differentially expressed genes must, by definition, come from the candidate set. Would it not be the case, therefore, that the significant intergene correlation would occur among candidate expression values that similarly respond to the change in treatment condition? Whereas two coexpressed genes would likely have correlated expression differentials, it is not this dependence that potentially causes false discoveries. To the extent that the correlation between genes *g*, *g*′ ∉ *ℍ*_0_ is a reflection of response to treatment conditions, the correlation is expected and informative. Correlation between truly expressed and unexpressed genes (possibly identified) reflects normal variations in expression unrelated to treatment condition. Correcting for such correlation (whether the effect is to increase or decrease the expression level) is important to the proper assessment of candidate *t*-scores. Moreover, the existence of the identified set, with its “noise only” interpretation of *t*-scores, makes possible this correction; ultimately resulting is reranked expression statistics.

To proceed, we must add some formality to the descriptions of the identified and candidate sets. Without loss of generality, we may assume that the complete set of genes is indexed so that

(3) $$\begin{array}{c} \left|t_{1}\right|\leq \left|t_{2}\right|\leq \cdots \leq \left|t_{G_{0}}\right|\leq \left|t_{G_{0}+1}\right|\leq \cdots \leq \left|t_{G}\right|, \end{array}$$

in which *G*_0_ is the number of genes declared null under the ZA. Then, genes with indices 1,2,…, *G*_0_ are assumed null and therefore comprise the identified set as defined above. Let us partition the set of *t*_*g*_ statistics into those corresponding to genes declared null under the ZA, {*t*_1_, *t*_2_,…, *t*_*G*_0__} and those for the remaining $G_{1}\overset{\mathrm{def}}{=}(G-G_{0})$ genes that continue to compete for the nonnull designation (the candidate set), {*t*_*G*_0_+1_, *t*_*G*_0_+2_,…, *t*_*G*_}. For convenience, express the vector **t** in terms of these two partitions:

(4) $$\begin{array}{c} t=\underset{{\left(t^{0}\right)}^{T}}{\underset{︸}{[\begin{array}{cccc} t_{1} & t_{2} & \cdots & t_{G_{0}} \end{array}}}\underset{{\left(t^{1}\right)}^{T}}{\underset{︸}{{\begin{array}{cccc} t_{G_{0}+1} & t_{G_{0}+2} & \cdots & t_{G} \end{array}]}^{T}}}=\left[\begin{array}{c} t^{0}\\ t^{1} \end{array}\right]. \end{array}$$

The random vector **t** has the following moments:

(5) $$\begin{array}{c} t=\left[\begin{array}{c} t^{0}\\ t^{1} \end{array}\right]\sim \left(\mu =\left[\begin{array}{c} \mu^{0}\\ \mu^{1} \end{array}\right],\Sigma =\left[\begin{array}{cc} \Sigma^{00} & \Sigma^{01}\\ \Sigma^{10} & \Sigma^{11} \end{array}\right]\;\right), \end{array}$$

in which $\mu^{i}\overset{\mathrm{def}}{=}ℰ\{t^{i}\}$ and $\Sigma^{ij}\overset{\mathrm{def}}{=}ℰ\{(t^{i}-\mu^{i}){\left(t^{j}-\mu^{j}\right)}^{T}\},\;\;\text{for}\;\;\;i,j=0,1$. Recall that the goal of the reranking process is to find a vector **τ** the elements of which represent a reordering of the elements of **t**, such that gene ranking represented by **τ** has better statistical power for detecting nonnull genes than that based on **t** itself. In the light of the newly defined notation in (5), we can more specifically say that **τ** represents a reevaluation and reordering of the *G*_1_ elements in **t** ^1^. The remaining elements of the revised vector, comprising the vector partition, say “${\tilde{t}}^{0}$,” are effectively set to zero since these genes are assumed to represent null genes. Recall that the *t*-scores in the **t** ^0^ vector (before processing) are assumed to represent noise variations around the nominal zero differential value for a null gene.

### 4.3. Theoretical Estimator of **τ**

Conditioned upon the random vector **t**, we seek a revised vector, ${\tilde{t}}^{1}(t)\equiv \tau$, in which the expression statistics for the candidate gene set (originally **t** ^1^) are uncorrelated with those of the identified set (originally **t** ^0^). There are many ways to derive the desired result, each with its own interpretation, but all, of course, ultimately equivalent. Whatever the approach is, it is expedient to remove the mean from the vector **t** ^1^ and work with the centered vector $t_{c}^{1}\overset{\mathrm{def}}{=}t^{1}-\mu^{1}$. The centered counterpart to the reranking vector, **τ**, will be denoted ${\tilde{t}}_{c}^{1}\overset{\mathrm{def}}{=}\tau -\mu^{1}$. The constant vector ***μ*** ^1^ will be returned to the result at the end. Recall that the mean of the vector **t** ^0^ is ***μ*** ^0^ = 0_*G*_0_×1_, so that “centering” is unnecessary for **t** ^0^ (throughout the paper, the notation 0_*I*×*J*_ denotes the zero matrix (vector) in the Cartesian space ℝ^*I*×*J*^).

To derive the desired expression for ${\tilde{t}}^{1}$, we adopt a simple approach based on well-known ideas from the theory of linear operators (e.g., [41–44]). Let us view the space of random vectors in (we are assuming *G*_1_ ≥ *G*_0_, or *p*_0_ ≤ 0.5. If the converse is true, simply reverse the roles of the ℝ^*G*_1_^ and ℝ^*G*_0_^ spaces in this development). ℝ^*G*_1_^ as a Hilbert space, *ℋ*, with inner product $\langle v,w\rangle \overset{\mathrm{def}}{=}ℰ\left\{v^{T}w\right\}$ for all **v**, **w** ∈ ℝ^*G*_1_^. The inner product induces a norm on *ℋ* given by $\;{||v||}_{2}\;\overset{\mathrm{def}}{=}\sqrt{\langle v,v\rangle }=\sqrt{ℰ\left\{v^{T}v\right\}}$, and, in turn, a metric

(6) d(v,w)=def||v−w||2=〈v−w,v−w〉ℰ{(v−w)T(v−w)}.

Now let *𝒮* be a closed subset of ℝ^*G*_1_^. Given some **v** ∈ ℝ^*G*_1_^, we wish to find closest element of *𝒮*, say $\hat{v}$, to **v**, in the sense that

(7) $$\begin{array}{c} \hat{v}=\underset{w\in 𝒮}{\text{argmin}\;}d\left(v,w\right), \end{array}$$

in which *d* is the metric in (6). The *Hilbert projection theorem* states the solution $\hat{v}$ exists and is unique. Moreover, $\hat{v}$ has the property that the vector difference between **v** and $\hat{v}$ is orthogonal to all vectors in the subspace *𝒮*; that is, $(v-\hat{v})\in {𝒮}^{⊥}$, in which *𝒮* ^⊥^ is the orthogonal complement to subspace *𝒮* in ℝ^*G*_1_^. In particular, random vector $\left(v-\hat{v}\right)$ is orthogonal to **v** ∈ *𝒮* ^⊥^ which means that $\langle (v-\hat{v}),v\rangle =0$ by definition. Thus, $ℰ\{{\left(v-\hat{v}\right)}^{T}v\}=0$, implying that $\left(v-\hat{v}\right)$ and **v** are stochastically orthogonal (uncorrelated if ***μ***_**v**_ = 0_*G*_1_×1_).

The Hilbert-space formulation provides the structure in which to achieve the desired decomposition of the random vector **t**_*c*_^1^ into components that are correlated, and uncorrelated, with **t** ^0^. Vector **t**_*c*_^1^ resides in the space ℝ^*G*_1_^. We seek a vector in a *G*_0_-dimensional subspace of ℝ^*G*_1_^ (because *G*_0_ is the dimension of **t** ^0^) that is close to **t**_*c*_^1^ (this will be the component of **t**_*c*_^1^ that is correlated with **t** ^0^). The problem at hand is only subtly different from one described in the generalities above. The difference is that we are not given a subspace “*𝒮*” in which to find an optimal vector; rather we are given only a *vector* **t** ^0^ which may reside in an uncountable number of subspaces of dimension *G*_0_. The goal will be to perform a linear operation on the given vector to “make it close” to **t**_*c*_^1^. In the process, we will inherently construct the subspace in which the optimal result resides. The subspace and resulting vector will be of dimension *G*_0_ because they are merely different representations of **t** ^0^ and its implicit initial subspace ℝ^*G*_0_^.

Given **t** ^0^, let us conceptualize a *G*_0_-dimensional subspace of ℝ^*G*_1_^, *𝒮*, consisting of all the (random) vectors {**v** : **v** = **F** **t** ^0^, **F** : ℝ^*G*_0_^ ↦ ℝ^*G*_1_^}. **F** represents some (yet unknown) linear operator of dimensions *G*_1_ × *G*_0_ and of rank *G*_0_. According to the Hilbert projection theorem, for a fixed **t** ^0^, there is a unique vector ${\hat{t}}_{c}^{1}=\hat{F}t^{0}\in 𝒮$, hence a unique linear operator, $\hat{F}$, such that

(8) d(tc1,F^t0)=argmin F∈ℝG1×G0rank(F)=G0d(tc1,Ft0)=argminF∈ℝG1×G0rank(F)=G0||tc1−Ft0||=argminF∈ℝG1×G0rank(F)=G0〈tc1−Ft0,tc1−Ft0〉=argminF∈ℝG1×G0rank(F)=G0ℰ{(tc1−Ft0)T(tc1−Ft0)}.

Since the metric is nonnegative, we may equivalently seek $\hat{F}$ that minimizes

(9) d2(tc1,Ft0)=ℰ{(tc1−Ft0)T(tc1−Ft0)}=ℰ{(tc1)Ttc1}−2ℰ{(tc1)TFt0}+ℰ{(t0)TFTFt0}.

Now, interpreting the expectation to be a mean-square average, we compute the gradient of *d* ^2^(**t**_*c*_^1^, **F** **t** ^0^) with respect to **F** (e.g., see [45]),

(10) $$\begin{array}{c} \nabla_{F}d^{2}\left(t_{c}^{1},Ft^{0}\right)=-2ℰ\left\{t_{c}^{1}{\left(t^{0}\right)}^{T}\right\}+2Fℰ\left\{t^{0}{\left(t^{0}\right)}^{T}\right\}, \end{array}$$

and set the result to 0_*G*_1_×*G*_0__, the solution to which is $\hat{F}$:

(11) $$\begin{array}{c} -ℰ\left\{t_{c}^{1}{\left(t^{0}\right)}^{T}\right\}+\hat{F}ℰ\left\{t^{0}{\left(t^{0}\right)}^{T}\right\}\equiv 0_{G_{1}\times G_{0}}. \end{array}$$

Recognizing that (recall (5)) *ℰ*{**t**_*c*_^1^(**t** ^0^)^*T*^} = Σ^10^ and *ℰ*{**t** ^0^(**t** ^0^)^*T*^} = Σ^00^, the solution becomes

(12) $$\begin{array}{c} \hat{F}=\Sigma^{10}{\left(\Sigma^{00}\right)}^{-1}. \end{array}$$

Now the random vector ${\hat{t}}_{c}^{1}\overset{\mathrm{def}}{=}\hat{F}t^{0}$ is as strongly linearly related to (correlated with) **t**_*c*_^1^ as is possible in the *G*_0_-dimensional subspace of ℝ^*G*_1_^ that is spanned by the *G*_0_ columns of operator $\hat{F}$. The correlation matrix relating ${\hat{t}}_{c}^{1}$ and **t**_*c*_^1^ is, say,

(13) Σ1^1=ℰ{t^c1(tc1)T}=ℰ{F^t0(tc1)T}=F^Σ01=Σ10(Σ00)−1Σ01.

Although $\Sigma^{\hat{1}1}$ is of dimension *G*_1_ × *G*_1_, it is clearly singular (rank *G*_0_) reflecting the inability of ${\hat{t}}_{c}^{1}$ to be linearly related to any component of **t**_*c*_^1^ in the subspace *𝒮* ^⊥^. In fact ${\hat{t}}_{c}^{1}$ is the component of **t**_1_ that embodies the potentially destructive correlation of the identified genes with the candidate genes. The decorrelated version of **t**_*c*_^1^ that we seek is therefore

(14) $$\begin{array}{c} {\tilde{t}}_{c}^{1}\left(t\right)=t_{c}^{1}-{\hat{t}}_{c}^{1}=t_{c}^{1}-\hat{F}t^{0}=t_{c}^{1}-\Sigma^{10}{\left(\Sigma^{00}\right)}^{-1}t^{0}. \end{array}$$

Note that ${\tilde{t}}_{c}^{1}$ is precisely the difference vector $t_{c}^{1}-\hat{F}t^{0}$ that is guaranteed by the Hilbert space theory to reside in *𝒮* ^⊥^ and therefore to be orthogonal, hence uncorrelated, with **t** ^0^. Finally, reinserting the mean value, ***μ*** ^1^, of the candidate vector, we have

(15) $$\begin{array}{c} \tau =t^{1}-\Sigma^{10}{\left(\Sigma^{00}\right)}^{-1}t^{0}. \end{array}$$

### 4.4. Sample Estimator of ${\tilde{t}}^{1}$

Estimates of the elements of Σ are required. For this purpose, we make several observations that are easily verified through simulation. Note that (15) does not require the covariance between *t*_*g*_ and *t*_*g*′_ when *g*, *g*′ ∉ *ℍ*_0_.

Let *γ*(*u*, v) and *ρ*(*u*, v) denote the scalar covariance and correlation, respectively, between real, scalar random variables, *u* and v:

(16) $$\begin{array}{c} \gamma \left(u,\text{v}\right)\overset{\mathrm{def}}{=}ℰ\left\{\left[u-ℰ\left(u\right)\right]\left[\text{v}-ℰ\left(\text{v}\right)\right]\right\}\\ \rho \left(u,\text{v}\right)\overset{\mathrm{def}}{=}ℰ\left\{u\text{v}\right\}=\gamma \left(u,\text{v}\right)+ℰ\left(u\right)ℰ\left(\text{v}\right). \end{array}$$

In these terms, we make the following observations.

**Observation 1 .**

If *g*, *g*′ ∈ *ℍ*_0_, then [3, 4]

(17) $$\begin{array}{c} \gamma \left(t_{g},t_{g^{\prime }}^{}\right)\approx \frac{\nu }{\nu -2}\;\rho \left(x_{g},x_{g^{\prime }}\right). \end{array}$$

**Observation 2 .**

If *g* ∈ *ℍ*_0_ and *g*′ ∉ *ℍ*_0_ (or conversely), then

(18) γ(tg,tg′) ≈νν−2  ×M2ρ(xg  ∣  1,xg′ ∣ 1)+M1ρ(xg  ∣  2,xg′  ∣  2)M1+M2

Equation (18) accommodates the possibility that the correlation between a null and a nonnull gene may change between treatment groups. If this does not occur, then (18) reduces to (17).

**Observation 3 .**

Furthermore, if *M*_1_ ≈ *M*_2_ (true for most microarray studies), then (18) simplifies to

(19) γ(tg,tg′)≈νν−2ρ(xg  ∣  1,xg′ ∣ 1)+ρ(xg  ∣  2,xg′  ∣  2)2.

Equations (17) and (19) suggest that we may use the sample covariance to estimate *γ*(*t*_*g*_, *t*_*g*′_):

(20) $$\begin{array}{c} \hat{\gamma }\left(t_{g},t_{g^{\prime }}^{}\right)\propto \frac{\sum_{m}{\overset{˘}{x}}_{gm}{\overset{˘}{x}}_{g^{\prime }m}}{\sqrt{\left(\sum_{m}{\overset{˘}{x}}_{gm}^{2}\right)\left(\sum_{m}{\overset{˘}{x}}_{g^{\prime }m}^{2}\right)}}, \end{array}$$

where ${\overset{˘}{x}}_{gm}$ denotes the expression level of the *g*th gene measured on the *m*th microarray after subtracting the average response within the treatment group to which *x*_*gm*_ belongs (*k* = 1 or 2). The scale factor *ν*/(*ν* − 2) cancels when the terms (Σ^00^)^−1^ and Σ^01^ are multiplied in (15), so that estimating *ν* is not required.

In the light of (20), (15) takes the practical form

(21) $$\begin{array}{c} {\tilde{t}}^{1}=t^{1}-{\hat{R}}^{10}{\left({\hat{R}}^{00}\right)}^{-1}t^{0}\overset{\mathrm{def}}{=}t^{1}-{\hat{R}}^{10}\;\hat{P}t^{0}, \end{array}$$

where ${\hat{R}}^{00}$ and ${\hat{R}}^{10}$ are the partitions (similarly to (5)) of $\hat{R}$, the sample correlation matrix of the gene expression matrix $\overset{˘}{X}$ (after removing the treatment effects). For notation compactness, we have defined $\hat{P}\overset{\mathrm{def}}{=}{\left({\hat{R}}^{00}\right)}^{-1}$. In principle, the set of elements in the vector ${\tilde{t}}^{1}$ of (15) embodies the gene-expression reranking in light of the compensatory measures taken to incorporate identifiability and to remove the effects of correlation. In practice, we rely on the *estimate* of  ${\tilde{t}}^{1}$ in (21).

## 5. Implementation

### 5.1. Gene Reranking Algorithm

A stepwise procedure for the gene reranking is given in Algorithm 1. The process begins by reindexing the genes based on their two-sample *t*-statistics (equation (3)). Then, based on the ZA, the first *G*_0_ genes are declared to satisfy *ℍ*_0_. In the experiments reported below, *p*_0_, the fraction of genes declared to be identifiable is set to ≈0.5 by default (the precise value is *p*_0_ = 6312/12625 for the database used which has *G* = 12625 total genes. See the second paragraph of Section 4.2 for an explanation). Although the choice 0.5 is somewhat arbitrary, this fraction is clearly justifiable and it has worked well empirically in the data sets tested. 

In order to nullify any genuine treatment differences, **X** is converted to $\overset{˘}{X}$ by subtracting each gene's average response within each treatment group. The sample correlation matrix $\hat{R}$ of $\overset{˘}{X}$ is subsequently computed. The critical step is to compute ${\tilde{t}}^{1}$ (equation (21)). The elements of  ${\tilde{t}}^{1}$ determine the gene ranking: gene *g* is ranked more highly than gene *g*′ if $\left|{\tilde{t}}_{g}^{1}\right|>\left|{\tilde{t}}_{g^{\prime }}^{\;1}\right|$ in which ${\tilde{t}}_{g}^{1}$ is the *g*th element of ${\tilde{t}}^{1}$. The first *G*_∗_ genes in the reranked list are reported as differentially expressed.

### 5.2. Numerical Stability and Computational Complexity

 Ordinarily, *M* ≪ *G*, so that the sample correlation matrix is severely rank deficient. A small quantity (typically 10^−10^) is added to the diagonal entries of $\hat{R}$ to make it invertible. After this augmentation, the algorithm above exhibits excellent numerical stability.

If implemented in a näive way, the matrix inversion to compute $\hat{P}={\left({\hat{R}}^{00}\right)}^{-1}$ in (21) would be a prohibitive operation in most computing environments, since microarray data sets may have several tens of thousand genes. Determining the rightmost product in (21), $\hat{P}t^{0}$, by solving the system of simultaneous linear equations ${\hat{R}}^{00}s^{0}=t^{0}$ for the vector **s** ^0^ = **P** **t** ^0^ is much faster than explicitly computing the matrix inverse $\hat{P}$ and forming the product. In particular, we can employ the Cholesky decomposition to exploit the fact that the matrix ${\hat{R}}^{00}$ is symmetric and positive definite (e.g., [46, Theorem 4.2.5]). MATLAB implementation uses the built-in function linsolve with appropriate settings, which, in turn, uses the highly optimized routines of LAPACK (http://www.netlib.org/lapack/).

The prostate cancer data [47] used in the experiments of Section 6 includes *G* = 12625 genes and *M* = 102 samples. For these data, the algorithm above implemented using MATLAB version R2006b on a computer with a 2.2 GHz dual-core AMD Opteron processor and 8 GB of RAM required ~40 seconds to report the final gene list. Similar implementation with explicit matrix inversions requires ~10 minutes. These times clearly indicate the *relative* benefit of avoiding the explicit matrix inversion, but the faster reporting time of ~40 sec should certainly not be interpreted as a lower bound for a problem of this scale. Indeed, workstations with 32 GB or more of RAM and with faster processors with eight or more processing cores are commercially available at modest costs. Of course, MATLAB is designed for modularity and ease of use, not computational efficiency. Dedicated, lower-level coding of the reranking steps, implemented on a faster machine with more parallelism could reduce the reranking time significantly. 

## 6. Experimental Results

### 6.1. Technical Comparisons

 The reranking method developed above is compared with two leading techniques, SAM (Significance Analysis of Microarrays [31]) and EDGE (Extraction and Analysis of Differential Gene Expression [14, 48]). SAM adds a small exchangeability factor *s*_0_ to the pooled sample standard deviation when computing the two-sample *t*-statistic:

(22) $$\begin{array}{c} t_{g}^{\prime }=\frac{{\overset{-}{x}}_{g\;\;|\;\;2}-{\overset{-}{x}}_{g\;\;|\;\;1}}{s_{g}+s_{0}}, \end{array}$$

whereas EDGE is based on a general framework for sharing information across tests. EDGE is reported to show substantial improvement in statistical power over five prominent techniques including SAM [14], the *t*/*F*-test [49, 50], the shrunken *t*/*F*-test [51], the empirical Bayes local FDR [40], and the *a posteriori* probability approach [52]. It is noteworthy that the reranking procedure developed here shows a significant performance improvement over EDGE in the experiments conducted. To determine the performance quality of various techniques, we focus primarily on the numbers of false positives, *𝔉*, and the corresponding FDR values, $\bar{𝔉}$, in the reported gene lists. Broadly speaking, the smaller the FDR, the better the technique.

### 6.2. Results

#### 6.2.1. Prostate Cancer Data

The primary experiments reported in this paper are based on the prostate cancer data from the work of Singh et al. [47]. This database includes expression data for *G* = 12625 genes on *M* = 102 oligonucleotide microarrays, comparing *M*_1_ = 50 healthy males with *M*_2_ = 52 prostate cancer patients. The purpose of the Singh study is to identify genes that might anticipate the clinical behavior of prostate cancer. The  .CEL files for the prostate study are publicly available at http://www-genome.wi.mit.edu/MPR/prostate. The general purpose of the present experiments is to compare performance of the reranking algorithm with the published state-of-the-art methods EDGE and SAM. The software RMAExpress [53] was used to obtain high-quality gene expressions from the posted data files. RMAExpress applied its in-built background adjustment; however, the quantile normalization was not used. To increase normality and stabilize across-group variances [54], each gene was represented in the final expression matrix **X** by the log of its expression level.

Comparative algorithm performance and insight into the inner workings of the reranking method required expression matrices for which truly differentially expressed genes were known *a priori*. Of course, in the nascent field of genomics, such knowledge is not available, and it is the very purpose of techniques like those discussed in this paper to seek such information. We approached this circular problem by using the prostate database to create test expression data with intergene correlation in **X** resembling that in the real microarray data. This was accomplished by first row standardizing the expression matrix from the prostate database. In particular, the true prostate matrix **X** was transformed to $\overset{˘}{X}$ by subtracting each gene's average response within each treatment group and by normalizing within group sample mean squares. That is, for the individual treatment groups (for *k* = 1, then for *k* = 2) and for each *g*:

(23) $$\begin{array}{c} M_{k}^{-1}\sum_{\overset{m=1}{\text{Group}\;}k}^{M}{\overset{˘}{x}}_{gm}=0,\;\;M_{k}^{-1}\sum_{\overset{m=1}{\text{Group}\;}k}^{M}{\overset{˘}{x}}_{gm}^{2}=1 \end{array}$$

in which ${\overset{˘}{x}}_{gm}$ is the (*g*, *m*) element of matrix $\overset{˘}{X}$. Each row represents one gene (and two conditions), so that with this transformation, all genes have equal energy and yet the same within group intergene correlation structure as the original **X**. Normalizing within-group sample mean squares to unity is not implemented in the reranking algorithm. The normalization is done here prior to any processing as a first step in creating an expression matrix with known differentiation of expression across groups for each gene, but with realistic (derived from real data) intergene correlation.

To generate a test data set from $\overset{˘}{X}$, its 102 columns were randomly divided into groups of *M*_1_ = 50 and *M*_2_ = 52. Next, *G*_+_ (*G*_−_) genes were randomly chosen for up- (down-) regulation by adding a positive (negative) offset *x*_+_ (*x*_−_) to the corresponding entries in group 2. The total number of truly differentially expressed genes is denoted (*G*_*δ*_ is to be contrasted with *G*_∗_, the number of genes determined by experimentation to be differentially expressed):

(24) $$\begin{array}{c} G_{\delta }=G_{+}+G_{-}. \end{array}$$

We also denote by $p_{\delta }\overset{\mathrm{def}}{=}G_{\delta }/G$ the proportion of *truly* differentially expressed genes, and, for future purposes, the ratio of the size of the desired gene-discovery list to the number of truly-differential genes, $p_{*\delta }\overset{\mathrm{def}}{=}G_{*}/G_{\delta }$. In the experiments, various choices of the simulation parameters,

(25) $$\begin{array}{c} {𝒫}_{\delta }\overset{\mathrm{def}}{=}\left\{p_{\delta },G_{+},G_{-},x_{+},x_{-}\right\}, \end{array}$$

were tested to represent a range of data scenarios encountered in practice. Also associated with each trial is a set of parameters characterizing the gene-discovery experiments, say,

(26) $$\begin{array}{c} {𝒫}_{e}\overset{\mathrm{def}}{=}\left\{p_{0},G,G_{*},M_{1},M_{2}\right\}. \end{array}$$

These parameter sets are detailed below.

In all experiments, results for the existing EDGE and SAM methods were obtained using the subroutines statex.r from the EDGE software package (http://www.genomine.org/edge/) and samr.r from the SAMR package (http://www-stat.stanford.edu/~tibs/SAM/), respectively. Both routines computed their native gene summary statistics given the matrix **X** and corresponding column labels. These statistics, in turn, were used to determine the top *G*_∗_ genes. Results based on reranking proceed from the steps outlined in Algorithm 1 with *G*_∗_ values corresponding to the largest $\left|{\tilde{t}}_{g}^{1}\right|$ scores of the reranked list.

Three experiments (cases) involving the prostate data are reported here. The first two cases were designed, by choice of the proportion of truly differential genes, *p*_*δ*_, to represent typical conditions of two general classes of gene-discovery problems. The third case was carried out to test robustness of the technique to small sample size.

**Case 1 (Small *p*_*δ*_).**

In the first case, *p*_*δ*_ is small, *p*_*δ*_ ~ 0.01–0.05, meaning that there are relatively few truly differentially expressed genes. The smaller *p*_*δ*_ is consistent with microarray investigations seeking genes that distinguish subtypes of cancer or diabetes, for example. The complete simulation parameter set for Case 1 is

(27) $$\begin{array}{c} {𝒫}_{\delta }^{1}=\{p_{\delta }=0.025,G_{+}={2G}_{-}=200,x_{\pm }=\pm 0.1\}, \end{array}$$

where *x*_±_ = ±0.1 means that *x*_+_ = +0.1 and *x*_−_ = −0.1. For numerical simplicity, we based the experiments on *G* = 12,000 of Singh's [47] gene expressions, so that *G*_*δ*_ = *p*_*δ*_ *G* = 300. Two sets of experiment parameters are used in Case 1, differing only in the size of the list of discovered genes.

*Subcase 1.1 (Small p*_*δ*_ *, Small p*_∗*δ*_ *). *In the first experiment, the parameter set is

(28) $$\begin{array}{c} {𝒫}_{e}^{1}=\{p_{0}=0.5,G=12000,G_{*}=100,M_{1}=50,M_{2}=52\}. \end{array}$$

The size of the gene-discovery list, *G*_∗_ = 100, is significantly smaller than *G*_*δ*_ = 300, or *p*_∗*δ*_ = *G*_∗_/*G*_*δ*_ = 1/3. In practice, a relatively small *G*_∗_ would be chosen to identify high-quality, class-distinguishing features for expression-profiling-based clinical diagnosis and prognosis, in which the goal is to build accurate classifiers and predictors. Whereas Singh et al. [47] build a classifier around only 16 of 12625 features, they discuss the need to include as many reliable features as possible.

**Figure 1:**
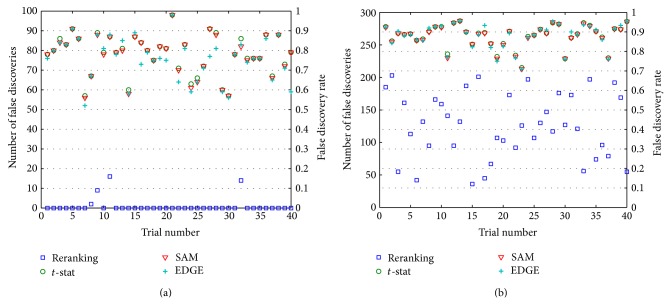
Results for two variations of Case 1 experiments. In both variations, the number of truly differential genes, *G*_*δ*_ = 300, and the “signal strength” (amount of up- or downregulation represented by *x*_+_ and *x*_−_) is weak. (a) Results for Subcase 1.1 in which the size of the gene-discovery list, *G*_∗_ = 100, is small relative to *G*_*δ*_ (*p*_∗*δ*_ = 1/3). The plot shows *𝔉* (left ordinate) and $\bar{𝔉}$ (right ordinate) values over 40 trials. (b) *𝔉* and $\bar{𝔉}$ results, plotted similarly to those of part (a) of the figure, for Subcase 1.2, in which *G*_∗_ = 300, large relatively to *G*_*δ*_ (*p*_∗*δ*_ = 1).

Figure 1(a) presents results for the test pair (*𝒫*_*δ*_^1^, *𝒫*_*e*_^1^) of Subcase 1.1. Remarkably, for 36 of 40 **X** matrices, the reranking procedure reports gene lists with no false discoveries at all, while the other techniques fail to obtain a single gene list with $\bar{𝔉}<0.5$. This result is typical of many “small *p*_*δ*_” experiments carried out with an array of parameter sets. In particular, the *quality* of the results notwithstanding (as measured by $\bar{𝔉}$, see below) the reranking strategy uniformly outperformed EDGE and SAM in every scenario.

In any rational detection algorithm built around a parametrized stochastic framework, it is possible to find regions of the parameter space in which performance deteriorates. In the “small *p*_*δ*_” gene identification problem, for a fixed *G* and *M*, increasing *G*_∗_ (more specifically, increasing the ratio *p*_∗*δ*_) or decreasing the “signal” magnitudes of either up-( *x*_+_) or down-(*x*_−_) regulation, all create increasing probabilistic risk of false discoveries, *𝔉*. As *G*_∗_ was allowed to approach *G*_*δ*_ in Case 1 experiment above, the performance of all methods, EDGE, SAM, and reranking, all deteriorated as measured by *𝔉*, yet the reranking approach remained consistently superior to the others according to this measure. “Better,” however, does not always mean “good.” For illustration, we report a second Case 1 experiment.

*Subcase 1.2 (Small p*_*δ*_ *, Large p*_∗*δ*_ *).* In this variation of Case 1 experiment, we take *G*_∗_ = *G*_*δ*_ = 300. This is still a “small *p*_*δ*_” situation, but with a “greedy” approach to gene discovery, an attempt to identify “all” genes (estimated to be) differentially expressed, *p*_∗*δ*_ = 1. Such a strategy would be employed in a microarray study designed to liberally identify a set of genes to be explored further—experimentally or computationally—to gain better understanding of underlying gene networks.

Figure 1(b) shows plots of the number of false positives, *𝔉*, over 40 data sets for Subcase 1.2 experiment. The corresponding FDR, $\bar{𝔉}=𝔉/G_{*}$, is shown on the secondary ordinate axis. With *p*_∗*δ*_ = 1 and with relatively weak differential expression “signals” (*x*_±_ = ±0.1), identifying a good gene lists is not an easy task, as evident from the results. Among all methods only reranking achieved sufficiently low values of $\bar{𝔉}$ to rescue a few **X** matrices, but, clearly, even reranking would not provide scientifically useful or reliable gene lists in this high-risk environment.

**Case 2 (Large *p*_*δ*_).**

The second case employs a larger *p*_*δ*_ ~ 0.1, typical of studies comparing healthy versus diseased cell activities. Simulation parameters for this case are

(29) $$\begin{array}{c} {𝒫}_{\delta }^{2}=\{p_{\delta }=0.1,G_{+}=\;G_{-}=600,x_{\pm }=\pm 0.02\}. \end{array}$$

Relative to Case 1, there are many more truly differentially expressed genes in Case 2 (increased by factor 4), thus decreasing the risk of false discoveries, especially for a small ratio *p*_∗*δ*_. This is akin to an increased prior probability of a differentially expressed gene in a Bayesian detection strategy. To further challenge the algorithms in the light of the “increased prior,” the up/downregulation of expression was made considerably weaker in Case 2 (reduced by factor five relative to Case 1), as in Case 1, two sets of experiment parameters were used in Case 2, differing only in the size of the list of discovered genes.

*Subcase 2.1 (Large p*_*δ*_ *, Small p*_∗*δ*_ *).* In the first Case 2 test, the experiment parameter set is given by

(30) $$\begin{array}{c} {𝒫}_{e}^{2}=\{p_{0}=0.5,G=12000,G_{*}=300,M_{1}=50,M_{2}=52\}. \end{array}$$

**Figure 2:**
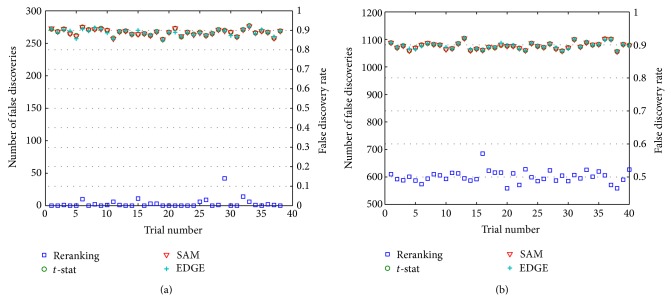
Case 2 *𝔉* (left ordinate) and $\bar{𝔉}$ (right ordinate) values over 40 trials with larger *p*_*δ*_ data. The number of truly differential genes, *G*_*δ*_ = 1200. The “signal” is very weak (*x*_±_ = ±0.02). (a) Subcase 2.1 results. *G*_∗_ = 300, small relative to *G*_*δ*_ (*p*_∗*δ*_ = 0.25). The plot shows the number of false positives, *𝔉*, over 40 data sets. The corresponding $\bar{𝔉}=𝔉/G_{*}$ is shown on the secondary ordinate axis. In spite of the relatively weak signal, reranking results in remarkably better performance than the standard approaches, and it produces the discovery list with no false positives in a majority of the 40 trials. (b) Subcase 2.2 results. *𝔉* and $\bar{𝔉}$ *G*_∗_ = 1200 (*p*_∗*δ*_ = 1).

The test pair (*𝒫*_*δ*_^2^, *𝒫*_*e*_^2^) represents a large *p*_*δ*_ proportion, but relatively small *p*_∗*δ*_ = 300/1200 = 0.25. The experimental results for this case over 40 trials are shown in Figure 2(a). In spite of the decreased signal strength, the reranking procedure produces no false discoveries in a vast majority of trials, similarly to the small *p*_*δ*_, small *p*_∗*δ*_, experiment of Subcase 1.1. EDGE and SAM consistently report a very large proportion of false discoveries (typically 250, or 90%).

*Subcase 2.2 (Large p*_*δ*_ *, Large p*_∗*δ*_ *).* A second Case 2 experiment was run to show the effects of “greedy” discovery lists, or large *p*_∗*δ*_ ratios. The parameters (*𝒫*_*δ*_^2^, *𝒫*_*e*_^2^) remain identical to those in Subcase 2.1 experiment, except that *G*_∗_ = 1200, so *p*_∗*δ*_ = 1. Results are shown in Figure 2(b). Like the large *p*_∗*δ*_ experiment in Subcase 1.2, the reranking approach significantly outperforms EDGE and SAM, with typically $\bar{𝔉}\sim 0.5$ for reranking and $\bar{𝔉}\sim 0.9$ for the standard methods. The sample variances for *𝔉* and $\bar{𝔉}$ are notably smaller in Subcase 2.2 trials relative to similar trials in Subcase 1.2. Also as in Subcase 1.2, reranking consistently outperforms the standard methods for large *p*_*δ*_ throughout the range 0 < *p*_∗*δ*_ ≤ 1. At some application-dependent point in this range, reranking, although “better,” is not sufficiently “good” at producing reliable discoveries. Clearly, in the present experiment in which *p*_∗*δ*_ = 1, the reranking result of typically $\bar{𝔉}\sim 0.5$ is not indicative of reliable gene discoveries. For a few trials, the rate drops as low as $\bar{𝔉}\sim 0.45$, but even this best-case rate implies ~540 false discoveries in the reported list of 1200 genes.

**Case 3 (Tests of Robustness to Small Sample Size).**

The number of microarray chips available to a study, *M*, is ordinarily quite small compared with the number of genes investigated, *G*. The gene discovery operation is therefore required to draw conclusions from a sparse sampling of the gene expressions. To gain some insight into the robustness of the gene-discovery methods to small sample sizes, variations on Case 2 (large *p*_*δ*_) experiments were repeated with the further stressor of a significant reduction in the sample space. *M*_1_ and *M*_2_ were each reduced to 20. So that observations could be more attributable to the sample size, *M*, the signal levels were increased back to Case 1 values of *x*_±_ = ±0.1. The simulation parameters used in Case 3, *𝒫*_*δ*_^3^, are identical to *𝒫*_*δ*_^2^ of (29). As in the previous cases, we ran two experiments, the first (Subcase 3.1) with a “conservative” gene discovery list, *G*_∗_ = 300 or *p*_∗*δ*_ = 0.25 and the second (Subcase 3.2) with a “greedy” gene discovery list of size *G*_∗_ = 1200 or *p*_∗*δ*_ = 1.

**Figure 3:**
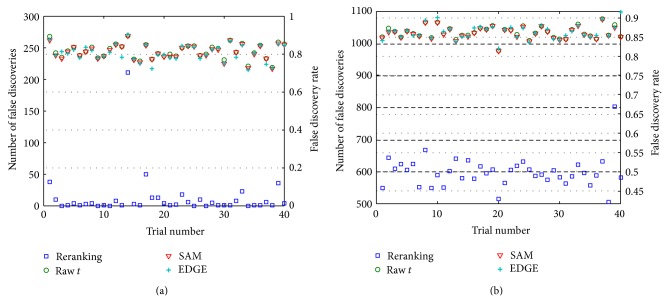
*𝔉* and $\bar{𝔉}$ values over 40 trials for Case 3 in which *G*_*δ*_ = 1200. (a) Subcase 3.1, *G*_∗_ = 300 (*p*_∗*δ*_ = 0.25); (b) Subcase 3.2, *G*_∗_ = 1200 (*p*_∗*δ*_ = 1).

To create the data set for Case 3, 20 columns per treatment group were chosen randomly from the original prostate cancer expression matrix **X**. The data generation process (including row standardization) detailed in Section 6.2.1 was then applied to the selected columns. Some compensation for the reduction in the number of samples is potentially present in the increased differential signal. The *𝔉* and $\bar{𝔉}$ values over 40 trials for the three methods are shown in Figure 3(a) for Subcase 3.1 and in Figure 3(b) for Subcase 3.2. Even with the significantly reduced sample size, the reranking process provides consistently superior performance with respect to existing methods. As in previous experiments, however, the better performance in the large *p*_∗*δ*_ experiment of panel (b) does not mean that the results are necessarily reliable or useful. Nevertheless, these results suggest that the reranking procedure increases power in the analysis of small sample data sets.

#### 6.2.2. Simulated Data

Before devising the test data setup of Section 6.2.1, the reranking method was tested on several simulated data sets. We discuss some of these simulation results that shed further light on the small sample behavior of the method.

Let us denote by **x**_*m*_ the *m*th column of a simulated expression matrix **X**. We assume that the random vector **x**_*m*_ is multivariate Gaussian with (stationary with index *m*) mean 0_*G*×1_ and covariance matrix Λ. Each such column represents *G* = 3226 genes, which, in their null expressions, are modeled by a covariance matrix Λ that introduces roughly the same amount of linear dependence as found in the BRCA data of [55]. We chose simulation parameters

(31) $$\begin{array}{c} {𝒫}_{\delta }=\left\{p_{\delta }=0.031,G_{+}=G_{-}=50,x_{\pm }=\pm 1\right\}, \end{array}$$

and the experiments were run with parameters

(32) $$\begin{array}{c} {𝒫}_{e}=\left\{p_{0}=0.5,G=3226,G_{*},M_{1}=M_{2}=10\right\}, \end{array}$$

for the two list sizes *G*_∗_ = 50 (*p*_∗*δ*_ = 0.5) and *G*_∗_ = 100 (*p*_∗*δ*_ = 1).

**Figure 4:**
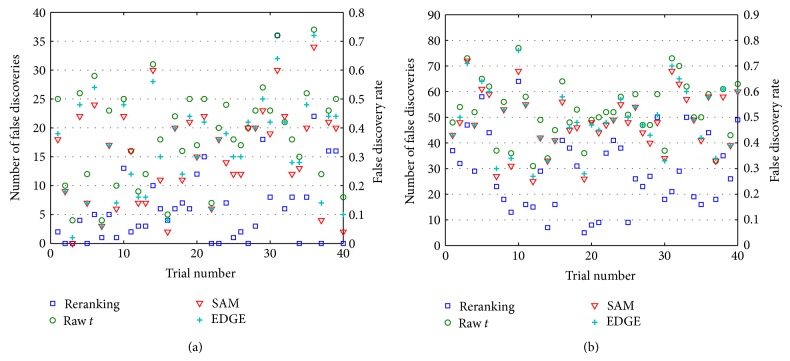
$\bar{𝔉}$ values over 40 trials using simulated expression data. Sample sizes are very small, *M*_1_ = 10, *M*_2_ = 10. (a) *G*_∗_ = 50 (*p*_∗*δ*_ = 0.5); (b) *G*_∗_ = 100 (*p*_∗*δ*_ = 1).

Figures 4(a) and 4(b) show plots of the *𝔉* and $\bar{𝔉}$ values over 40 trials for *G*_∗_ = 50 and *G*_∗_ = 100, respectively. With a smaller *M*, preeminence of the reranking method scales down. Nevertheless, for 26 out of 40 simulated **X** realizations, reranking achieves an FDR $\bar{𝔉}<0.15$ and for 30 of 40 trials, $\bar{𝔉}<0.25$.

Table 1 shows results for some of the **X** realizations for *G*_∗_ = 100 (Figure 4(b)). Shown are the largest 100 values of $\left|{\tilde{t}}_{g}^{1}\right|$ and each corresponding original *t*_*g*_ with concomitant rank. Even in this challenging case, the results indicate favorable aspects of the reranking procedure. First, it is noteworthy that reranking results in *𝔉* = 22 false discoveries in the list of 100 genes, whereas *𝔉* = 68 when raw *t* statistics are used. Further, all but two of the false discoveries reported by reranking received an even higher ranking by *t*-statistics. On the other hand, 46 of the correct discoveries by reranking would not have appeared in the list of 100 genes reported by *t*-statistics. Results like these were observed repeatedly in our data analysis. Consistent with the results of the prostate data studies, the reliability and utility of all techniques lessen as *p*_∗*δ*_ → 1, yet, reranking persistently outperforms the other methods.

It is notable that, with a smaller *M*, SAM outperforms EDGE and the use of raw *t*-scores. This is not entirely surprising as a smaller *M* can make the noise in the per gene pooled variance *s*_*i*_ (and possibly the equivalent quantity in the EDGE algorithm) more prominent. SAM mitigates this issue in some measure by using the exchangeability factor *s*_0_ to adjust the effective pooled variance [31].

## 7. Discussion and Conclusions

In most microarray data, there are at least three resources that can be used to advantage: (i) identifiability, (ii) parallel structure, and (iii) intergene correlation itself. Analysis in papers by Efron [56, 57] suggests this view of the rich information structure inherent in the data. In this light, reranking can be viewed as exploiting more than correlation as a means of sharing information across tests, as it also involves identifiability.

Limited time and resources often require biomedical researchers to work on only a small number of “hot (gene) prospects.” Even under such highly conservative conditions, however, misleading results can occur, as is evident in the results of Figures 1–4. For all their expert development and statistical power, even state-of-the-art tools like SAM and EDGE can report spurious gene lists. The extra statistical power of reranking promises to further guard against anomalous results that can have serious consequences for the study of gene function, causation, and interaction.

In summary, this paper has reported the development and testing of a novel framework for the detection of differential gene expression. The framework exploits identifiability—the fact that in most microarray data sets, a large proportion of genes can be identified *a priori* as nondifferential—to reduce the correlation in the expression data for the remaining gene candidates. When applied to the widely used two-sample *t*-statistic approach, this viewpoint yielded a simple differential analysis technique which requires as inputs only a gene expression matrix, related two-sample labels, and the size of desired output gene-list *G*_∗_. The method was tested on data constructed from the prostate cancer database of Singh et al. [47] and some simulated data. Compared with SAM [31], EDGE [14], and the raw *t*-statistic approach itself, reranking shows substantial improvement in statistical power. As is the case with all published techniques, the reranking process' power tends to increase considerably with an increase in the number of microarray samples. However, even for small sample sizes, performance was significantly better than the alternatives in the experiments conducted here.

## Figures and Tables

**Algorithm 1:**
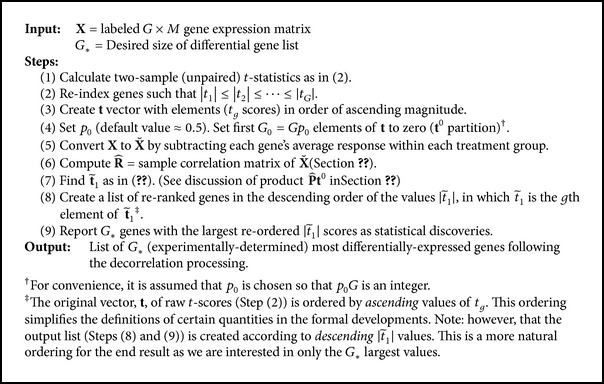
Steps in the gene re-ranking procedure.

**Table 1:** Top  100  ${\tilde{t}}_{g}^{1}$ scores determined by re-ranking. Corresponding *t*_*g*_ scores and their ranks are also shown. The results are for some of the **X** realizations from Figure 4(b). Data representing false discoveries are printed in boldface. ${\#}_{{\tilde{t}}_{g}^{1}}≝$ rank based on ${\tilde{t}}_{g}^{1}$ score, #_*t*_*g*__≝ rank based on raw *t*_*g*_ score.

${\#}_{{\tilde{t}}_{g}^{1}}$	${\tilde{t}}_{g}^{1}$	*t*_*g*_	#_*t*_*g*__
1–50			

1	4.22	5.87	1
2	−4.17	−5.55	2
3	−3.93	−4.26	5
4	−3.74	−4.12	7
5	−3.58	−4.49	4
6	−3.49	−3.34	28
7	−3.45	−4.25	6
8	3.35	3.87	10
9	−3.33	−3.20	35
10	3.33	3.77	13
11	3.25	3.42	25
12	−3.16	−2.18	260
13	3.14	4.54	3
14	3.10	2.87	65
15	−3.08	−3.54	17
16	−3.07	−2.80	80
17	3.06	3.49	20
18	3.02	2.29	213
19	−2.99	−3.34	27
20	−2.93	−3.13	38
21	−2.92	−2.92	57
22	2.86	3.26	31
23	−2.83	−2.82	74
24	2.82	2.37	180
25	−2.81	−2.13	276
**26**	**2.81**	**3.48**	**21**
27	−2.79	−3.01	47
28	2.70	2.87	64
29	−2.66	−3.15	37
**30**	**−2.58**	**−3.85**	**11**
31	−2.56	−2.84	71
32	−2.55	−1.72	524
33	−2.54	−2.63	106
34	−2.54	−2.69	98
35	2.53	2.30	209
36	2.48	2.45	148
37	−2.47	−2.29	212
38	−2.46	−3.21	33
39	−2.43	−2.44	154
40	2.43	2.71	94
41	2.40	2.86	66
42	−2.34	−2.60	115
43	−2.34	−2.98	50
**44**	**−2.33**	**−3.80**	**12**
45	−2.32	−2.06	306
46	2.29	1.81	444
47	−2.27	−1.17	1110
48	2.26	1.97	347
**49**	**2.24**	**3.75**	**14**
**50**	**2.20**	**3.88**	**9**

51–100			

**51**	**2.18**	**3.45**	**23**
**52**	**2.17**	**3.05**	**42**
53	2.16	2.80	82
54	−2.15	−2.57	122
55	2.15	1.96	357
56	−2.14	−1.47	751
57	2.13	2.25	229
58	2.13	1.77	486
59	2.13	1.44	785
60	2.12	2.14	273
61	−2.11	−1.48	744
**62**	**2.10**	**1.80**	**453**
63	2.09	2.60	114
64	−2.09	−2.05	312
65	2.09	2.70	96
66	2.09	2.23	237
67	2.08	2.34	188
68	−2.08	−2.24	232
69	−2.06	−2.53	130
70	−2.04	−2.11	283
**71**	**−2.04**	**−2.95**	**54**
**72**	**−2.03**	**−3.08**	**40**
73	−2.02	−2.30	210
**74**	**−2.01**	**−3.67**	**15**
75	2.00	2.62	109
76	−1.98	−2.38	171
77	1.98	1.43	795
78	1.96	1.69	549
79	−1.95	−1.47	746
80	1.95	1.95	361
**81**	**1.95**	**2.81**	**77**
82	−1.94	−1.41	813
**83**	**1.94**	**3.40**	**26**
84	1.94	1.30	948
**85**	**−1.94**	**−3.27**	**30**
86	−1.93	−1.11	1190
87	−1.93	−1.37	872
**88**	**−1.93**	**−3.44**	**24**
**89**	**−1.92**	**−3.07**	**41**
90	−1.92	−1.50	726
**91**	**1.90**	**3.62**	**16**
**92**	**−1.90**	**−2.82**	**75**
93	1.89	1.25	1007
**94**	**1.87**	**3.89**	**8**
**95**	**−1.86**	**−3.49**	**19**
**96**	**−1.86**	**−2.08**	**300**
97	1.85	1.20	1074
**98**	**−1.83**	**−2.90**	**60**
99	1.83	1.39	833
100	−1.82	−1.95	367
